# Biallelic *COA7*-Variants Leading to Developmental Regression With Progressive Spasticity and Brain Atrophy in a Chinese Patient

**DOI:** 10.3389/fgene.2021.685035

**Published:** 2021-07-12

**Authors:** Rui Ban, Zhimei Liu, Masaru Shimura, Xiao Tong, Junling Wang, Lei Yang, Manting Xu, Jing Xiao, Kei Murayama, Matthias Elstner, Holger Prokisch, Fang Fang

**Affiliations:** ^1^Department of Neurology, National Center for Children’s Health, Beijing Children’s Hospital, Capital Medical University, Beijing, China; ^2^Institute of Human Genetics, Helmholtz Zentrum München, Munich, Germany; ^3^Department of Metabolism, Chiba Children’s Hospital, Chiba, Japan; ^4^Department of Neurology, Beijing New Century International Children’s Hospital, Beijing China; ^5^Department of Neurology, School of Medicine, Technical University of Munich, Munich, Germany; ^6^Institute of Human Genetics, Technische Universität München, Munich, Germany

**Keywords:** *COA7/RESA1*, biallelic variant, COX assembly factor, mitochondrial disease, nervous system disorder

## Abstract

**Objective:**

The cytochrome c oxidase assembly factor 7 (*COA7)* gene encodes a protein localized to mitochondria that is involved in the assembly of mitochondrial respiratory chain complex IV. Here, we report the clinical, genetic and biochemical analysis of a female patient with suspected mitochondrial disorder and novel variants in *COA7*, that presented with a considerably different phenotype and age of onset than the five *COA7* patients reported to date.

**Methods:**

We performed trio-exome sequencing in the affected patient and both parents. To verify the pathogenicity of the detected variants in *COA7*, mitochondrial enzyme activities and oxygen consumption rate were investigated in fibroblasts of the patient and her parents.

**Results:**

A Chinese girl was referred at 9 months of age with a history of developmental delay and regression since 3 months of age. In the following months, she lost previously acquired skills and developed progressive spasticity of the lower extremities. Trio-exome sequencing revealed compound heterzygous variants in *COA7* (c.511G > A/p.Ala171Thr and c.566A > G/p.Asn189Ser). Functional validation experiments revealed isolated complex IV deficiency and a significantly reduced mitochondrial respiration rate in patient-derived fibroblasts.

**Interpretation:**

Hitherto, characteristic features of *COA7* patients were described as slowly progressing neuropathy and spinocerebellar ataxia, starting at the toddler age and progressing into adulthood. In contrast, our patient was reported to show developmental delay from 3 months of age, which was found to be due to a rapidly progressive encephalopathy and brain atrophy seen at 9 months of age. Unexpectedly, the genetic investigation revealed a *COA7-*associated mitochondrial disease, which was confirmed functionally. Thus, this report broadens the genetic and clinical spectrum of this heterogeneous mitochondriopathy and highlights the value of the presented unbiased approach.

## Introduction

Mitochondrial diseases are multisystem disorders that arise as a result of mitochondrial respiratory chain dysfunction caused by pathogenic variants in either the mitochondrial DNA or nuclear DNA known to encode mitochondrial proteins (Spinazzola and Zeviani, 2009). Although the clinical spectrum of mitochondrial diseases is broad, the majority of affected individuals present with prominent neurologic and myopathic features.

Cytochrome c oxidase (COX), also referred to as complex IV (CIV), is made up of fourteen subunits that catalyze the transfer of electrons from ferrocytochrome c to molecular oxygen. To date, 26 nuclear genes encoding either CIV subunits (*n* = 9) or CIV assembly factors (*n* = 16) have been described (Stenton and Prokisch, 2020). Cytochrome c oxidase assembly factor 7 (*COA7*), also known as C1orf163/SELRC1, encodes a soluble mitochondrial protein. The COA7 protein is localized in the intermembrane space (IMS) of mitochondria and has a putative function in the assembly of CIV (Kozjak-Pavlovic et al., 2014). So far, only five affected individuals with biallelic mutations in *COA7* have been reported worldwide. One patient presented with leukoencephalopathy, the other four presented with spinocerebellar ataxia with axonal neuropathy (Martinez Lyons et al., 2016; Higuchi et al., 2018).

In the present study, we identified novel biallelic *COA7* variants in one affected Chinese girl with developmental regression, progressive leukoencephalopathy and cerebral atrophy. Functional tests in patient-derived fibroblasts indicated an isolated CIV defect and reduced mitochondrial respiratory rate. As such, this case report broadens the clinical and genetic spectrum of *COA7*-related mitochondrial disease.

## Patient and Methods

### Case Report

The female patient was the first child of healthy non-consanguineous Chinese parents, born at full-term by natural vaginal delivery after an uneventful pregnancy. Anthropometric data, postnatal adaptation and neonatal course were unremarkable.

Delayed motor development became apparent starting at 3 months of age by missed milestones. The girl could not lift her head until the third month after birth, could not play with both hands until the fourth month, could not grasp objects actively until the fifth month and could not turn over until the seventh month, but she was able to follow people or a moving object with her eyes and call papa in the fifth month. At ninth months of age, she could not yet sit unassisted.

As a result of the reported developmental delays, the girl was referred to our hospital at 9 months of age. She was able to roll over but was unable to sit independently. Her parents reported that she had made some sounds at the age of 5 months but had lost this ability recently as well as some motor skills following frequent infections. On neurological examination, she displayed hypertonia of the upper extremities with fisting and trembling of both hands. She had a normal muscle tone in the legs and normal reflexes at this point. Constant and progressive developmental regression was seen after an episode of diarrhea and fever. She lost the ability to roll over, presented head lag and made poor eye contact. Her feeding had become increasingly difficult. Physical examination at 11 months of age showed marked muscular hypertonia and hyperreflexia, spasticity, and tonic dystonia in lower extremities. During the following 5 months, the spasticity worsened, repeatedly induced by intestinal infection but was reasonably controlled with baclofen treatment. She is currently 2 years old.

**FIGURE 1:**
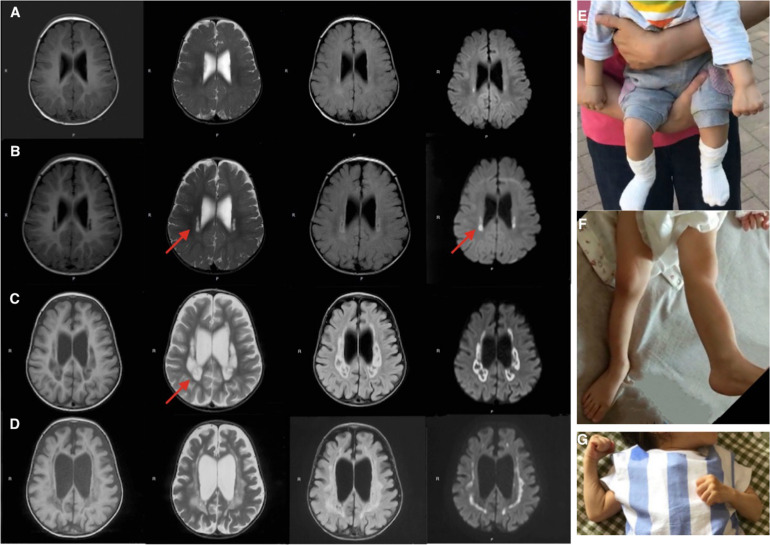
Brain MRI (T1-weighed) showing progressive brain atrophy. MRIs of the patient taken at **(A)** 9, **(B)** 11, **(C)** 16, and **(D)** 21 months of age. Note the high T2 signal in the periventricular regions on restriction diffusion weighed images **(B,C)**. **(E,F)** Progressive muscular hypertonia at 11 and 16 months old. **(G)** Partially controlled hypertonia after baclofen treatment at 2 years old.

Brain MRIs at age 9, 11, 16, and 21 months showed progressive brain atrophy (see Figures 1A–D) with extended diffusion restriction in the bilateral periventricular areas. The patient’s progressive muscular hypertonia at age 11 (Figure 1E) and 16 (Figure 1F) months was partially controlled by 24 months (Figure 1G) with baclofen.

Cardiac ultrasound showed enlargement of left atrial diameter (anteroposterior diameter thickness 21 mm), thickened ventricle septa (thickness 8 mm) and left posterior wall (thickness 8 mm), mild mitral and tricuspid regurgitation and a small pericardial effusion.

Extensive diagnostic screening showed elevated serum CK (488 U/L, normal range 5–220 U/L), mildly elevated alanine aminotransferase (ALT, 54 U/L, normal range 7–40 U/L), aspartate aminotransferase (AST, 65 U/L, normal range 13–45 U/L), and lactate dehydrogenase (LDH, 274 U/L, normal range 50–240 U/L). Serum lactate was elevated (2.98 μmol/L, normal range 0.5–2.2 μmol/L). Metabolic values were normal in both blood and urine.

### DNA Extraction, Mitochondrial DNA and Whole-Exome Sequencing

Genomic DNA from the family was extracted from peripheral whole blood samples using the TIANamp Blood DNA Kit (DP348) according to the manufacturer’s instructions.

The genomic DNA was fragmented, ligated with the paired-end adaptor, amplified and purified. All human mitochondrial genes, exons and the 50 bp bases in their adjacent introns were captured by SeqCap EZ Med Exome Enrichment Kit (Roche NimbleGen). The DNA library was performed post-capture amplification and purification, and then sequenced using the Illumina HiSeq sequencing platform. To get the coverage and mean read depth of the target regions, the sequence data was aligned to the human genome reference (hg19) and variants-calling were used with NextGene V2.3.4 software. The mean read depth was 151.24 × and it reached 20 × for 97.95% of the target sequences.

Variants were screened as followed: (1) Preference to the variants related to the diseases, small INDEL, canonical splice sites and missense variants. (2) Minor allele frequency in normal populations < 5% (except for the known MAF ≥ 5% pathogenicity). (3) Preference to the variants in HGMD, ClinVar. (4) Preference to the variants in OMIM. The variants of pathogenicity were in accordance with the standards and guidelines for the interpretation of sequence variants published by ACMG in 2015 with HGVS nomenclature. For the *COA7* gene, we used NM_023077 as the reference sequence for the transcript.

### Prediction of the Possible Impact of an Amino Acid Substitution

Mutations of *COA7* were analyzed for deleteriousness by the Polyphen-2^1^ and PROVEAN program^2^. Sequence alignment of the COA7 protein in different species was performed using the Clustal OMEGA program.

Sequences of wild-type COA7 were submitted to the Swiss-Model server^3^ for modeling in automated mode on template 6deh (LpnE). The crystal structure of the Legionella effector protein LpnE(73–375) was solved to 1.75Å resolution (Voth et al., 2019). Both proteins belong to the SEL1-like repeat family with e-value at 9.7e-69 and 3.7e-15 for LpnE and COA7, respectively^4^ and can be evolutionary linked by Pfam HMM and phylogenetic tree mapping (Schreiber et al., 2014; Mistry et al., 2021). With a GMQE score of 0.6, 25% sequencing identity (BLOSUM62 62 62 matrix), 89.2% coverage, and local QMEAN scores of 0.813 and 0.779 for the A171 and N189 variants, the model reached a meaningful quality. Structures and stability effects of mutations were built and analyzed using the FoldX modeling suite v5. Structures were visualized in PyMOL (PyMOL Molecular Graphics System V 2.4.0a0).

### Cell Culture

Skin fibroblasts from the family and control fibroblasts (fHDF, Toyobo) were cultured in Dulbecco’s modified Eagle medium (DMEM; Gibco^®^, Life Technologies, Thermo Fisher Scientific) in the presence of 1% penicillin/streptomycin and 10% fetal bovine serum (both Gibco^®^, Life Technologies, Thermo Fisher Scientific) at 37°C and 5% CO_2_.

### Oxidative Phosphorylation (OXPHOS) Enzyme Assay by Spectrophotometry

Activities of OXPHOS complexes I, II, II + III, III, IV, and the mitochondrial marker enzyme citrate synthase were assayed in isolated mitochondria obtained from skin fibroblasts, as described previously (Kirby et al., 1999; Frazier and Thorburn, 2012; Ogawa et al., 2017). The experiment was performed in duplicates and values represent the average of two measurements. Enzyme activities of complex I-IV are presented as the percentage of mean normal control activity relative to citrate synthase enzyme activity.

### Oxygen Consumption Rate (OCR) Measurement

OCR was measured in cultured fibroblasts derived from the family, using the XF96 Extracellular Flux Analyzer (Seahorse Bioscience, Billerica, MA, United States). Samples were prepared as previously reported (Kremer and Prokisch, 2017; Ogawa et al., 2017). In brief, control and patient’s family fibroblasts were seeded in at least 16 wells of two XF96 cell culture microplates at a density of 20,000 cells/well in 80 μL growth medium and incubated at 5% CO_2_ at 37°C overnight. The following day, the growth medium was replaced with 160 μL of 25 mM glucose medium or 10 mM galactose medium, and the microplate was placed into a CO_2_-free incubator at 37°C for 60 min before measurement. After measurement of the basal OCR, 10 μM oligomycin, 4 μM carbonyl cyanide phenylhydrazone (FCCP), and 20 μM rotenone/antimycin were added sequentially, and OCR was recorded after each addition (Kremer and Prokisch, 2017). The maximum respiration rate (MRR) corresponds to the OCR after FCCP injection minus rotenone-insensitive OCR. MRR was expressed as percentages relative to the average of controls, in which a reduction to < 71.6% is considered to represent a significant decline (Ogawa et al., 2017).

## Results

### Sequencing Analysis

**FIGURE 2:**
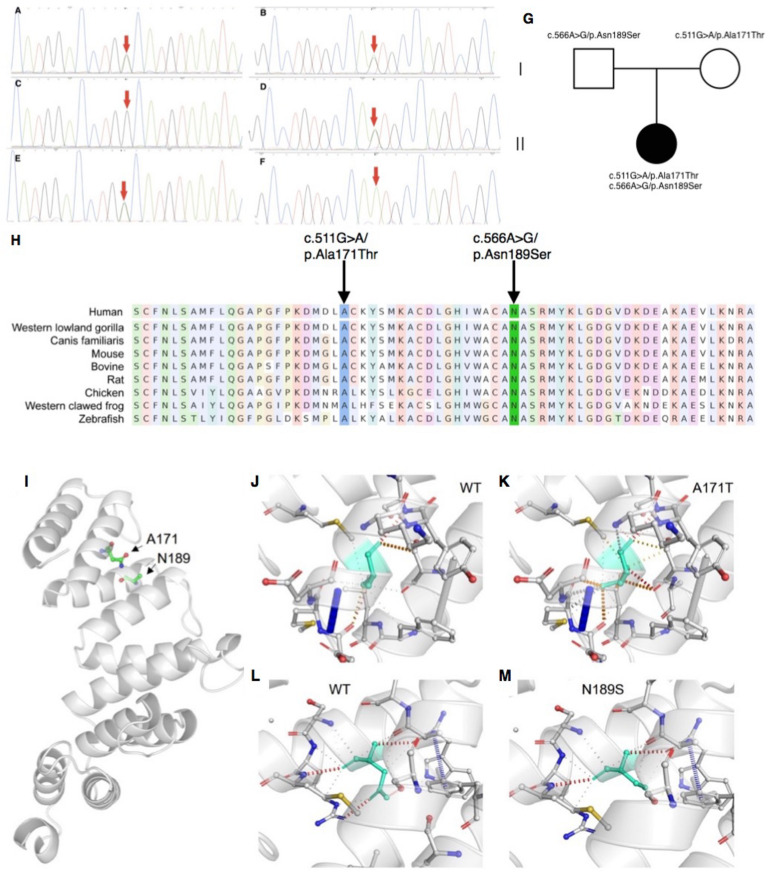
Sanger sequencing, conservation analysis and homology modeling. **(A–G)** Sanger sequencing and pedigree showing the segregation of the variants in the family. **(A,B)** The patient carries the c.511G > A/p.Ala171Thr and c.566A > G/p.Asn189Ser mutations. **(C,D)** The patient’s father carries the c.566A > G/p.Asn189Ser but not c.511G > A/p.Ala171Thr mutation. **(E,F)** The patient’s mother carries the c.511G > A/p.Ala171Thr but not c.566A > G/p.Asn189Ser mutation. **(G)** Segregation of variants within the family. **(H)** Conservation analysis showing both sites of the missense mutations were conservative among different species. **(I–M)** The impact of *COA7* missense mutations on protein structure. **(I)** COA7 as a ribbon representation; residues A171 and N189 are drawn with a ball-and-stick representation. **(J,K)** Detailed view of the environments of A171, A171T, N189 and N189S where the residues are colored in light-green and represented as sticks, possible interactions between neighbor atoms are indicated with dashed lines, dark red for hydrogen bonds, green for hydrophobic contacts, and deep plink for carbonyl contacts.

Mitochondrial DNA sequencing did not reveal any pathogenic variants. Whole-exome sequencing detected two missense variants in *COA7*: the maternally derived c.511G > A/p.Ala171Thr, and the paternally derived c.566A > G/p.Asn189Ser (Figures 2A–G), which were confirmed by Sanger sequencing.

### Variant Interpretation and Conservation Analysis

Neither variants were found in ClinVar nor HGMDpro databases, while in the GenomAD database, c.511G > A/p.Ala171Thr was absent but c.566A > G/p.Asn189Ser showed a total allele count of 3 with an extremely low heterozygote allele frequency of 0.00001061. The PROVEAN program predicted both mutations discovered in the *COA7* gene to be deleterious (PROVEAN score −3.855 and −4.818, respectively), and Polyphen-2 predicted both to be probably damaging with scores of 0.996 and 1, respectively. Conservation analysis showed both sites of the missense mutations were conservative among different species (Figure 2H). Homology modeling showed that both of the two mutations localized at the central part of the Sel1-like α helices repeats (Figure 2I). To determine whether the mutations could affect the structure of COA7, thermodynamic stability was estimated using FoldX. Moderate destabilizing effects were predicted for both Ala171Thr and Asn189Ser, with ΔΔG = 2.46 and 1.42 kcal/mol, respectively. The destabilizing effect of the Ala171Thr mutation is due to the substitution of the non-polar hydrophobic side-chain of alanine that increases the polar solvation energy and the van der Waals energy clashes (Figures 2J,K). The loss of a hydrogen bond in the Asn189Ser mutation is the major cause of the protein’s destabilization (Figures 2L,M).

### Oxidative Phosphorylation (OXPHOS) Enzyme Activity and Oxygen Consumption Rate (OCR)

OXPHOS enzyme activity showed isolated decreased CIV activity in the patient’s derived fibroblasts when compared to that of her parents, who had normal control values (Table 1). The OCR analysis of our patient’s fibroblasts showed a significantly lower maximum respiration rate (MRR, % of control) in glucose- and galactose-containing medium (46.1 and 45.7% of control; Table 2).

**TABLE 1:** Mitochondrial enzyme activities in cultured fibroblasts derived from the patient and her parents.

	**mU/U CS**	**Reference range**	**(mean ± SD)**	**% of control**
**Patient**				
C I	370.0	237–618	(433 ± 85)	85.4
C II	571.0	364–826	(545 ± 97)	104.9
C II + III	279.4	298–795	(526 ± 133)	53.1
C III	126.0	32–143	(94 ± 27)	133.6
C IV	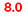	13–35	(19 ± 5)	41.1
**Father**				
C I	325.0	237–618	(433 ± 85)	75.1
C II	512.0	364–826	(545 ± 97)	94.1
C II + III	302.0	298–795	(526 ± 133)	57.3
C III	172.0	32–143	(94 ± 27)	182.6
C IV	24.4	13–35	(19 ± 5)	126.0
**Mother**				
C I	227.0	237–618	(433 ± 85)	52.5
C II	436.0	364–826	(545 ± 97)	80.1
C II + III	499.1	298–795	(526 ± 133)	94.8
C III	126.0	32–143	(94 ± 27)	133.7
C IV	17.8	13–35	(19 ± 5)	91.8

*Activities (absolute values) of complexes I, II, III, and IV are presented as milliunits per unit (mU/U) citrate synthase (CS). Relative activity is calculated as percentage of mean activity of normal controls. Red/bold shows values indicating a deficiency of the respective complex activity. C I, complex I; C II, complex II; C III, complex III; C IV, complex IV.*

**TABLE 2:** Oxygen consumption rate (OCR) in cultured fibroblasts derived from the patient and her parents.

		**OCR glucose (% of control)**	**OCR galactose (% of control)**
Patient	Basal	89.9 ± 3.7	73.3 ± 6.3
	Oligomycin	115.9 ± 2.3	94.6 ± 4.0
	MRR	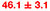	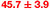
Father	Basal	117.5 ± 3.0	125.3 ± 9.3
	Oligomycin	158.0 ± 2.0	155.6 ± 3.3
	MRR	102.4 ± 3.6	83.1 ± 4.5
Mother	Basal	94.6 ± 2.6	91.6 ± 5.1
	Oligomycin	99.6 ± 1.3	104.9 ± 6.2
	MRR	82.7 ± 3.6	72.8 ± 3.4

*OCR values are represented as [%] of control. Shown are basal and oligomycin-inhibited respiration rates in glucose or galactose media. Maximum respiration rate (MRR) is calculated from FCCP-uncoupled respiration minus rotenone/antimycin-insensitive oxygen consumption (non-mitochondrial). Data represents an average of ≥ 12 technical replicates ± SD. Bold/Red text indicates values outside of the normal range.*

## Discussion

Mitochondrial disorders are a clinically and genetically heterogeneous group of progressive multisystem disorders. Symptoms often begin in childhood and include neurological or developmental problems such as encephalopathy, epilepsy, neuropathy and movement disorders (Lightowlers et al., 2015).

Mitochondrial diseases are caused by mutations in mitochondrial DNA or nuclear DNA sequences that encode proteins relevant for mitochondrial protein biosynthesis, assembly and proper function. Mitochondrial protein functions can be affected by these mutations in many ways; they not only impair enzymatic activities, but can also lower protein stability, hamper assembly into multimeric protein complexes, or abrogate protein transport into mitochondria (Habich and Riemer, 2019). The *COA7* gene, also known as *1orf163* and *RESA1*, encodes a mitochondria-localized protein that is involved in the assembly of mitochondrial CIV, which is the last enzyme component of the mitochondrial respiratory chain (Martinez Lyons et al., 2016).

**FIGURE 3:**
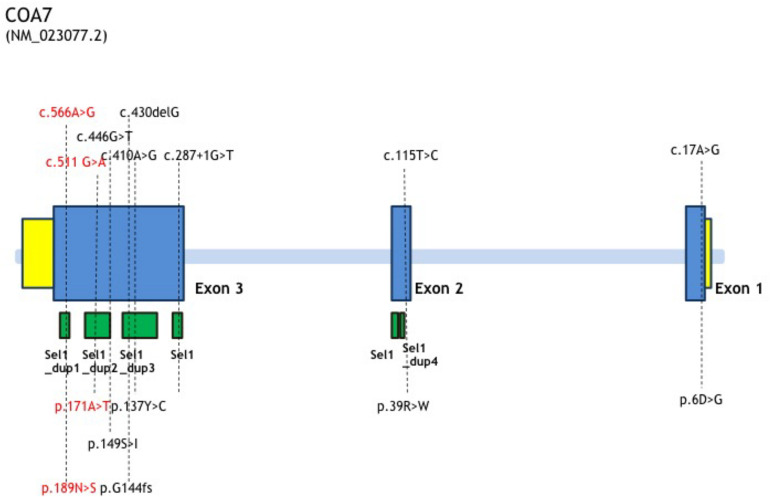
*COA7* mutation status, gene structure of affected amino acid residues. Gene structure of *COA7* mutations in 5 patients. The newly found mutations of this study are shown in red.

In this study, we report the first Chinese patient with recessive mutations in *COA7*, characterized by developmental delay and regression, progressive spasticity and progressive brain atrophy as well as white matter changes. To date, only five cases with *COA7*-related mitochondrial disease have been reported in the literature, all of which were adult patients. In 2016, the first patient was reported by Martinez Lyons et al. (2016), characterized by severe ataxia, peripheral neuropathy and mild cognitive impairment with leukoencephalopathy and spinal cord atrophy, the other 4 patients were characterized by axonal-type motor and sensory neuropathy with ataxia together with cerebellar atrophy (Higuchi et al., 2018), which were similar to the first patient. The clinical phenotypes and genotypes of all the published patients carrying *COA7* recessive mutations (together with our patient) are listed in Table 3 and Figure 3. Our patient shows an overlapping phenotype with leukoencephalopathy and brain atrophy but does not yet show any ataxia or neuropathy. Additionally, the infection-related regression and progressive spasticity reported in our patient as well as the mild cardiomyopathy have not been described previously. Furthermore, our patient presented much earlier than the other published patients.

**TABLE 3:** The clinical phenotypes of patients with biallelic mutations in COA7.

**No.**	**1**	**2**	**3**	**4**	**5**	**This patient**
Gender	M	M	F	M	M	F
Variants	c.410A > G/p.Y 137C and c.287 + 1G > T	c.17A > G/p.D6G &c.17A > G/p.D6G	c.115C > T/p.R39W and exon 2 deletion	c.17A > G/p.D6G and c.446G > T/p.S149I	c.17A > G/p.D6G and c.430delG/p.G144fs	c.511G > A/p.A171 T and c.566A > G/p.N189S
Age of onset(y)	1	< 5	4	15	< 5	3 m
Age last alive(y)	19	63	21	28	27	2
Developmental delay	Y-psychomotor	NA	NA	NA	N	Y
Developmental regression	NA	NA	NA	NA	N	Y
Intelectual disability	Y-mild	NA	Y	NA	N	N
Dysarthria	Y	Y	Y	Y	NA	N
Muscle weakness	Y, with muscle atrophy	NA	Y-distal	Y-distal	Y	N
Hypertonia	NA	NA	NA	NA	NA	Y
Ataxia	Y	Y	Y	Y	Y	N
Neuropathy	Y	Y	Y	Y	Y	N
Elevated CSF/serum lactate	Y-serum	Y-serum	N	Y-CSF	N	Y
White matter abnormality	Y	NA	Y	NA	Y	Y
Cerebral atrophy	NA	Y	NA	NA	NA	Y
Cerebellar atrophy	NA	Y	Y	Y	Y	N
Thin spinal cord	Y	NA	NA	Y	NA	Y
References	Martinez Lyons et al., 2016	Higuchi et al., 2018	Higuchi et al., 2018	Higuchi et al., 2018	Higuchi et al., 2018	This study

Despite the difference in clinical presentations, the fact that COA7 protein is known to be highly expressed in the cerebral cortex, caudate and heart muscle, and *COA7* RNA is known to be highly expressed in the cerebral cortex (Human Protein Atlas^5^), is consistent with our patient’s clinical manifestations.

Genetically, the study of the family indicated that *COA7* variants co−segregated the phenotype in-keeping with an autosomal−recessive inheritance. Both variants were missense mutations and were predicted to be deleterious or probably damaging in pathogenicity prediction tools. Furthermore, these two mutations were either absent or at an extremely low allele frequency rate in different public databases. Conservation analysis showed both mutations were highly conserved among species. We also predicted the 3D homology models of COA7, which indicated that the mutations could affect the mitochondrial function due to their protein destabilization effects.

Functionally, we conducted OXPHOS enzyme assay and calculated OCR in the patient and her parents. The respiratory chain enzyme assay of skin fibroblasts showed isolated CIV deficiency in the patient but was normal in her parents. The OCR assay showed similar segregation in her family, demonstrating a definite impairment in mitochondrial function in the patient only. In previously reported patients, lentiviral-mediated expression of recombinant wild-type *COA7* cDNA in the patient fibroblasts led to the recovery of the defect in COX activity and restoration of normal COX amount (Martinez Lyons et al., 2016). In 2014, Kozjak-Pavlovic et al. (2014) found that knockdown of *C1orf163* leads to reduced levels of OXPHOS complexes proteins, with the reduced activity of complex I and the strongest defects in the assembly of CIV in C1orf163-depleted cells, which was reconfirmed by Martinez Lyons et al. (2016). In Higuchi’s study, two patients with *COA7* recessive mutations manifested as isolated deficiency of CIV in fibroblasts, with one patient having low activity of complex I in fibroblasts (Higuchi et al., 2018). The variable effects of the mutation on OXPHOS enzyme activity and the clinical features may have been caused by a different interaction between COA7 and MRC complexes, possibly because of the difference in the site of mutation in the *COA7* gene (Higuchi et al., 2018). Isolated CIV deficiency in our patient is consistent with most of the other patients with *COA7* biallelic mutations.

COA7 is a 231αα long mitochondrial protein present in animals, containing five Sel1-like tetratricopeptide repeat sequences, which are likely to interact with partner proteins (Martinez Lyons et al., 2016).

The original characterization of the protein localized COA7 in the intermembrane space (Kozjak-Pavlovic et al., 2014). Data by Martinez Lyons et al. (2016) implied its location in the mitochondrial matrix. Higuchi et al. (2018) determined that COA7 was targeted to mitochondria in general, without analyzing its submitochondrial localization. More detailed biochemical and immunofluorescence analyses by Mohanraj et al. showed convincingly that COA7 is targeted to the intermembrane space (Kremer and Prokisch, 2017). Moreover, COA7 requires the mitochondrial IMS import and assembly pathway for efficient accumulation in the IMS, with pathogenic variants of COA7 being imported slower than the wild-type protein. COA7 proteins that are not imported are degraded in the cytosol by the proteasome, as proteasome inhibition rescued both the mitochondrial localization of COA7 and the CIV activity in patient−derived fibroblasts. Intriguingly, the authors propose that proteasomal inhibition could serve as a novel therapeutic target for mitochondrial diseases caused by protein import defects (Mohanraj et al., 2019).

In summary, pathogenicity prediction, conservation analysis, homology modeling, mitochondrial respiration complex enzyme deficiency and decreased OCR together support that the two mutations reported herein are novel causative variants in the *COA7* and responsible for the phenotype of development regression, progressive spasticity, brain atrophy and mild cardiomyopathy. Nevertheless, most published cases had a considerably later onset and milder phenotype, so that this case report expands the known genomic as well as the phenotypic spectrum of *COA7* related mitochondrial disease.

## Data Availability Statement

The datasets for this article are not publicly available due to concerns regarding participant/patient anonymity. Requests to access the datasets should be directed to the corresponding authors.

## Ethics Statement

The studies involving human participants were reviewed and approved by the Beijing Children’s Hospital. Written informed consent to participate in this study was provided by the participants’ legal guardian/next of kin.

## Author Contributions

RB carried out the clinical assessment, reviewed the literature, and wrote the draft manuscript. ZL performed the OXPHOS enzyme activity and OCR experiments. KM and MS supervised enzyme activity and OCR experiments. KM, MS, and ZL analyzed enzyme activity and OCR experiments. XT cultured the cells and provided imaging. JW liaised with patient’s parents and provided phenotype data. LY assisted with treatments. MX aided the interpretation of the case. JX performed the trio exome sequencing. ME, HP, and FF supervised the project and helped to writing the manuscript. All authors provided critical feedback.

## Conflict of Interest

The authors declare that the research was conducted in the absence of any commercial or financial relationships that could be construed as a potential conflict of interest.

## Footnotes

1 http://genetics.bwh.harvard.edu/pph2

2 http://provean.jcvi.org/index.php

3 https://swissmodel.expasy.org/

4 http://www.ebi.ac.uk/interpro/entry/InterPro/IPR006597/

5 http://www.proteinatlas.org
